# Modeling of Chemoperfusion vs. Intravenous Administration of Cisplatin in Wistar Rats: Adsorption and Tissue Distribution

**DOI:** 10.3390/molecules25204733

**Published:** 2020-10-15

**Authors:** Galina Kireeva, Stepan Kruglov, Mikhail Maydin, Ekaterina Gubareva, Elena Fedoros, Ekaterina Zubakina, Natalya Ivanenko, Marina Bezruchko, Nikolay Solovyev

**Affiliations:** 1 N.N. Petrov National Medical Research Center of Oncology, 197758 Saint Petersburg, Russia; galinakireyeva@mail.ru (G.K.); stepkaspb93@mail.ru (S.K.); mikhail.maidin@pharminnotech.com (M.M.); gubareva1984@gmail.com (E.G.); elenafedoros@gmail.com (E.F.); 2 Institute of Toxicology, Federal Medical Biological Agency, 192019 Saint Petersburg, Russia; mihailova_katya@inbox.ru (E.Z.); nbivanenko@mail.ru (N.I.); bezrucko@mail.ru (M.B.); 3 Institute of Technology Sligo, Ash Lane, F91 YW50 Sligo, Ireland

**Keywords:** *cis*-diamminedichloridoplatinum (II), hyperthermic intraperitoneal chemoperfusion, Wistar rats, platinum protein binding, intravenous injection, inductively coupled plasma mass spectrometry

## Abstract

Hyperthermic intraperitoneal chemoperfusion (HIPEC) is an established form of locoregional chemotherapy of peritoneum tumors. However, its efficacy and safety status remain a controversy, partially, due to scarce data on pharmacokinetics and toxicity profile of drugs under HIPEC. In the current study, 24 female Wistar rats were randomly assigned to receive cisplatin as HIPEC (*n* = 12, 20 mg/kg) or intravenously (i.v., *n* = 9, 4 mg/kg). The subgroups of three animals were used for the initial, intermediate, and late phases of the pharmacokinetic assessment. The animals were sacrificed on days 1 and 5. Blood, liver, kidney, and ovaries were evaluated for platinum content. Histological and immunohistochemical evaluation was undertaken in the liver and kidney. A trend for higher blood plasma platinum levels was observed for HIPEC compared to i.v. Significantly lower (*p* < 0.001) relative platinum binding to the proteins was observed in HIPEC animals compared to the i.v. administration. A five-fold higher concentration of cisplatin in HIPEC resulted in a ca. 2.5-fold increase in total blood platinum and ca. two-fold increase in blood ultrafitrable platinum (“free” Pt). Immunohistochemistry revealed higher kidney and liver damage after i.v. administration of cisplatin compared to HIPEC, although a five-fold higher dose of cisplatin was applied in HIPEC. Together with relatively lower absorption to the systemic circulation in HIPEC, higher protein binding is probably the primary reason for lower observed toxicity in HIPEC animals.

## 1. Introduction

Platinum (Pt)-based drugs are a g group of cytotoxic agents with DNA binding capacities [1], suppressing the replication [2]. These properties of platinum complexes were first described in the late 1960s with the discovery of the cytostatic properties of cisplatin, *cis*-dichlorodiammineplatinum (II) [3]. Since then, numerous transition metal complexes were explored as prospective antitumor agents [4]. Nevertheless, only a few compounds are approved for clinical use [5], including cisplatin (a parent compound of the family) [6], carboplatin, oxaliplatin, and nedaplatin (in some countries) [7]. The development of other platinum-based drugs, first of all, that of carboplatin and oxaliplatin, was stimulated by the side effects of cisplatin, including nephrotoxicity, ototoxicity, and peripheral nervous system disorders [7,8,9]. The main shortcomings of platinum-based cytostatic agents are associated with their insufficient selectivity for malignant cells, severe side effects, and drug resistance for some tumor-types [10,11].

Hyperthermic intraperitoneal chemoperfusion (HIPEC) combined with cytoreductive surgery is used for certain types of cancers affecting the peritoneum [12]. The concept of this method is to provide a short-term circulation of high doses of chemotherapeutic drugs in the peritoneal cavity. This provides higher efficacy against residual tumor nodes with no significant increase in toxicity, due to limited systemic absorption of the drugs in HIPEC [13,14]. Besides, hyperthermia per se serves as an additional therapeutic factor [15]. Cisplatin is amongst the most commonly used drugs for HIPEC, since its limitations under standard intravenous (i.v.) injection and infusion protocols (first of all, pronounced nephrotoxicity) are largely alleviated under HIPEC treatment [16,17].

The clinical application of HIPEC for almost 30 years has proven its efficacy against certain tumor types (pseudomyxoma peritonei and malignant peritoneal mesothelioma) [18,19]. Most convincing evidence in favor of HIPEC regimes with cisplatin (100 mg/m^2^) for locally advanced ovarian cancer, was recently provided in a randomized controlled trial by van Driel et al. The authors achieved significant benefit in median overall survival for HIPEC compared to surgery alone (45.7 months vs. 33.9 months) [20]. However, HIPEC remains a controversy in patients with locally advanced colorectal and ovarian cancer, as patient outcomes vary considerably. The expected outcomes are in the range from 50–60% 5 year survival to no improvement, compared to surgery combined with systemic chemotherapy [21,22]. One of the reasons for such controversy may be related to the fact that the underlying mechanisms and pharmacokinetics of the drugs under HIPEC are understudied. There is still no definite understanding of the distribution of chemotherapeutic agents after HIPEC and no comprehensive data on the toxicity profile of HIPEC. Such data are lacking from both preclinical and clinical studies, to the best of the authors’ knowledge. In this study, we explored the pharmacokinetics, including platinum protein binding, and toxicity of HIPEC with cisplatin in a rat model in comparison to the i.v. administration.

## 2. Results

### 2.1. Pharmacokinetics of Cisplatin in HIPEC Compared to i.v. Administration

#### 2.1.1. Pharmacokinetics Curves for HIPEC vs. i.v. Administration

**Figure 1:**
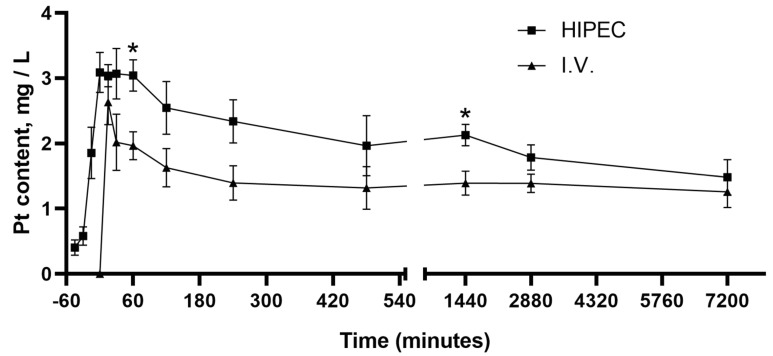
Platinum concentration in blood plasma (mean ± SEM) in rats after the perfusion (Hyperthermic intraperitoneal chemoperfusion: HIPEC) with cisplatin (20 mg/kg b.w.) and intravenous (i.v.) injection (4 mg/kg b.w.) from the beginning of the HIPEC (−45 min) and i.v. injection (0 min) up to 5 days. *—*p* < 0.05 compared to i.v. (Mann–Whitney U rank test). For the number of animals per time point, please refer to the main text (Section 4.2).

The pharmacokinetic curves for chemoperfusion and i.v. injection of cisplatin in doses of 20 mg/kg and 4 mg/kg, respectively, are shown in Figure 1. These doses were established as maximum tolerated doses for both routes in Wistar rats with transplanted ovarian cancer, which was described elsewhere [23]. A trend for higher blood plasma platinum levels is observant for the HIPEC compared to i.v.; however, it reaches statistical significance only for the time points of 60 and 1440 min (*p* < 0.05). The calculated areas under curves (AUCs) are as follows: AUC_(0–120)_ = 246.56 (h × mg × kg^−1^) and AUC_(0–120)_ = 167.65 (h × mg × kg^−1^) for HIPEC and i.v., respectively.

#### 2.1.2. Recovery of Perfusate

We studied the retention of platinum in the body of the rats after the HIPEC treatment. The data are shown in Table 1. Relative retention of platinum after HIPEC was ca. 39%.

#### 2.1.3. Platinum Distribution in Organs and Tissue

**Figure 2:**
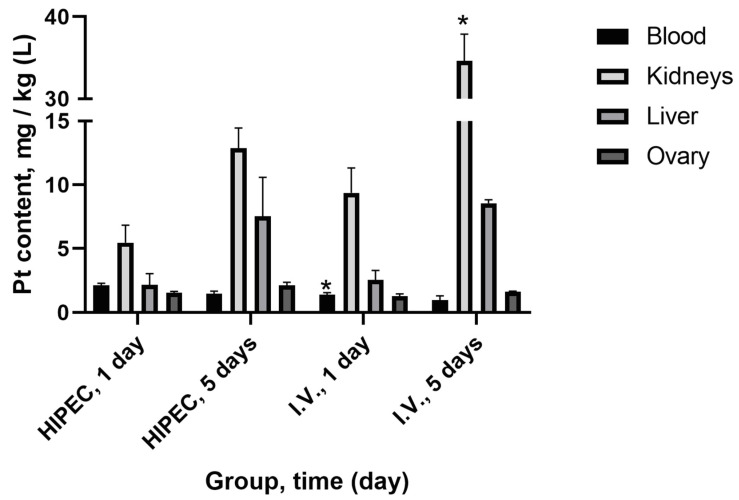
Platinum concentration in blood and organs (mean ± SEM) in rats after perfusion (HIPEC) with cisplatin (20 mg/kg b.w., *n* = 3) and intravenous (i.v.) injection (4 mg/kg b.w., *n* = 3) after 1 and 5 days. *—*p* < 0.05 compared to HIPEC (Mann–Whitney U rank test).

Platinum tissue distribution was studied on days 1 and 5 for both protocols of cisplatin administration (Figure 2). Significant differences in platinum level (*p* < 0.05) between HIPEC and i.v. were observed for blood on day 1—higher for HIPEC and for kidney on day 5—higher for i.v., although the total administered dose of platinum was significantly lower in the i.v. administration group.

### 2.2. Ultrafiltration

The results of the quantification of “free”/ultrafiltrable (10 kDa cutoff) platinum are shown in Table 2. The permeability through the filter (the ratio of platinum concentration in ultrafiltrate to whole blood platinum concentration) and relative protein binding of platinum (as a percent of retained platinum) were also calculated.

Significantly lower (*p* < 0.001) relative platinum binding to the proteins in systemic bloodstream was observed in HIPEC treated animals compared to the i.v. administration of cisplatin. Additionally, a five-fold higher concentration of cisplatin in HIPEC resulted in a ca. 2.5-fold increase in total blood platinum and a ca. two-fold increase in blood ultrafitrate platinum concentration (“free” Pt).

### 2.3. Histology and Immunohistochemistry

#### 2.3.1. Kidney

**Figure 3:**
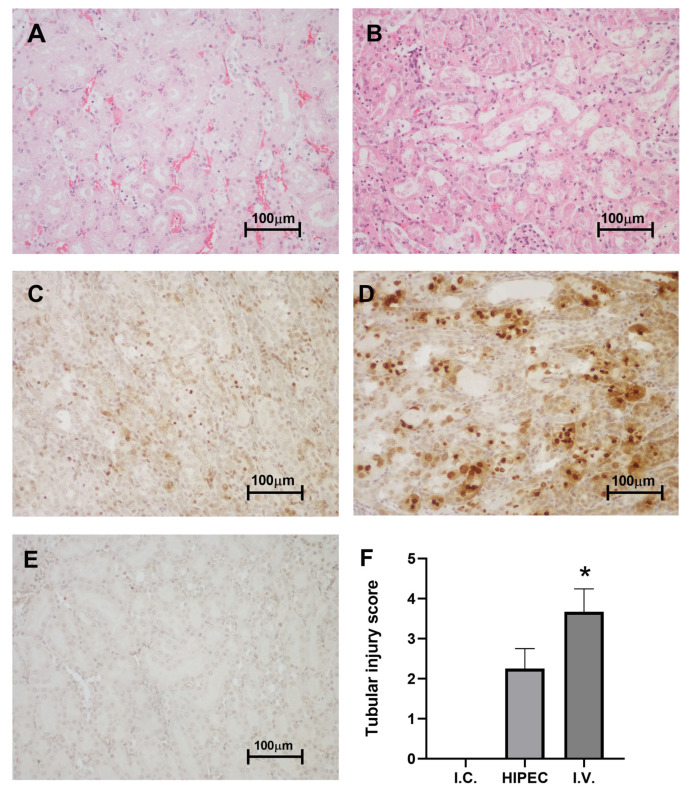
(**A**)—HIPEC, hematoxylin–eosin (H&E), ×200, (**B**)—intravenous (i.v.) injection of cisplatin, H&E, ×200, (**C**)—HIPEC, gammaH2AX, ×200, (**D**)—i.v., gammaH2AX, ×200, (**E**)—intact control (I.C.), gammaH2AX, ×200, (**F**)—average tubular injury score. *—*p* < 0.05 compared to I.C. (Kruskal–Wallis H rank test).

Tubular injury index (number of cystic tubules) was assessed for hematoxylin–eosin (H&E) stained sections. In the intact control group, no signs of tubular injury were observed. In the intravenous cisplatin injection group (i.v.), tubular injury index was high and reached 3.67 ± 0.33 (*p* = 0.013 compared to intact control), while in the HIPEC group it was lower −2.25 ± 0.25, *p* > 0.05 compared to i.v. or intact control (Figure 3).

GammaH2AX immunostaining revealed single positive nuclei in the control group, moderate staining in HIPEC and intense staining in i.v. injection group. Stained nuclei localized mostly in the proximal and distal tubules.

#### 2.3.2. Liver

**Figure 4:**
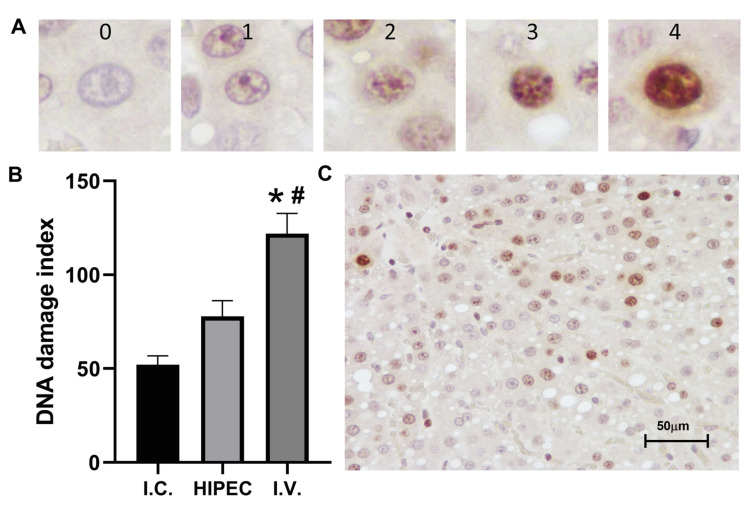
DNA damage in liver tissue (anti-gammaH2AX staining) (**A**)—hepatocytes nuclei types/classes (**0**—no visible staining, **1**—one or several small foci, **2**—more than 3 foci or weak staining of most chromatin, **3**—moderate staining of most or total chromatin, **4**—intense staining), (**B**)—DNA damage index, (**C**)—representative photomicrograph of liver tissue (i.v. group). *—*p* < 0.05 compared to the control group, #—*p* < 0.05 compared to the HIPEC group (Kruskal–Wallis test).

DNA damage index in hepatocytes (Figure 4) was significantly higher in i.v. group (121.9 ± 10.7) in comparison to control (52.0 ± 4.9, *p* = 0.001) and HIPEC group (77.8 ± 8.4, *p* = 0.034). Thus, less systemic toxicity of HIPEC is associated with a lower rate of DNA damage in peripheral tissues. GammaH2AX staining was not assessed in the ovaries as the intensity and distribution of stained nuclei varied among different follicle types and did not differ markedly between treatment groups (data not shown).

## 3. Discussion

Cisplatin, when administered as HIPEC, has a more favorable toxicity profile compared to systemic administration, which has been one of the rationales to perform HIPEC instead of i.v. injections/infusions [24,25,26]. The tolerance of the subjects to the administration of higher doses of drugs compared to the systemic route is also among the arguments in favor of HIPEC [24,27,28]. For cisplatin, AUC i.p./AUC i.v. ratio equals 12, which is beneficial in HIPEC settings [29]. Indeed, it has been the most widely used drug for HIPEC in different types of tumors. In ovarian cancer, the common dose of cisplatin used varies from 50 to 75 mg/m^2^ for the i.v. route [30]. There is no recommended dose of cisplatin for HIPEC; however, in the majority of studies and protocols, it exceeds 75 mg/m^2^, ranging from 75 to 200 mg/m^2^ [16,31,32].

Hyperthermia is known to mediate the cytotoxicity of antitumor agents [33,34]. There were several reports on platinum anticancer drugs having a considerable synergetic effect under mild hyperthermia (39–41 °C) [15,35,36]. Piché et al. studied the alteration of oxaliplatin concentration in the abdominal cavity wall and systemic blood flow in rats, depending on the perfusate’s temperature (37, 40 and 43 °C). They observed a proportional increase in oxaliplatin levels in both the tissue and blood with the rise of the temperature [37]. In contrast to that, Zeamari et al. reported no increase in cisplatin intake by the abdominal tumor cells under hyperthermia compared to normothermia [38]. However, clinical outcomes were not evaluated in this study.

In our study, we also performed HIPEC with five times the dose of cisplatin for i.v. administration, which has been established as safe in our previous research [23]. HIPEC resulted in higher Pt concentration in the circulatory system compared to i.v. administration. Several studies described a similar effect when systemic absorption of drugs during perfusion was increased, due to vasodilation caused by hyperthermia [39,40]. However, it was not associated with higher toxicity of HIPEC. There are limited data on the pharmacokinetics of intraperitoneal administration of cisplatin in rats with no data on the pharmacokinetics in HIPEC in experimental animals, to the best of the authors’ knowledge. It was shown that a single intraperitoneal injection of cisplatin at a dose of 7.5 mg/kg is followed by fast absorption of the drug with the peak of blood plasma concentration of 12.2 µg/mL, which was reached 5 min after the administration [41]. In our study, we observed a fast peak, related to platinum absorption to the systemic bloodstream after the start of the HIPEC treatment (Figure 1). The animals tolerated a much higher dose of cisplatin of 7.7 mg/kg b.w., calculated from the platinum retention experiment (Table 1). This was considerably higher than that for the i.v., where only 4 mg/kg b.w. cisplatin could be applied. The reason for comparable platinum levels observant in the bloodstream between the HIPEC and i.v. under considerably higher exposure in the HIPEC group may be attributed to the partial deposit of cisplatin in the abdominal adipose tissue for HIPEC rats. However, this notion is neither supported nor disregarded by the late elimination phase of the pharmacokinetic curve and requires further insight.

The protein binding testing using “free” (ultrafiltration) platinum assay was undertaken to elucidate the lower observed toxicity, first of all, in the kidney (Figure 3) and liver (Figure 4). Ultrafiltration is a well-accepted approach to evaluate plasma protein binding of the substances in vivo [42], also widely used in metal toxicology [43,44,45]. Interestingly, we showed considerably higher (nearly two-fold) relative protein binding for the HIPEC treatment compared to i.v. injection of cisplatin. Lower nephrotoxicity was also corroborated by the tissue distribution of platinum (Figure 2), indicating relatively low kidney platinum accumulation after the HIPEC treatment compared to i.v. administration. Together with relatively lower absorption to the systemic circulation in the HIPEC, higher protein binding is probably the primary reason for lower observed toxicity in HIPEC animals. The prospects for further studies may be related to the investigation of platinum distribution in abdominal adipose tissue of HIPEC animals and the study of specific platinum protein binding under HIPEC administration to elucidate the reasons for enhanced protein binding.

## 4. Materials and Methods

### 4.1. Animals

Wistar female rats weighing 220–280 g, 4-month-old (Rappolovo animal facility, Leningrad region, Russia) were used in the studies. Study protocols were approved by the Local Ethics Committee of the N.N. Petrov National Medical Research Center of Oncology (Protocol No. 11, dated 21 September 2018). The animals were acclimated for at least 14 days before the experiments commenced. The animals were maintained on a 12:12 light–dark cycle at 21 ± 2 °C with 50 ± 20% average humidity and had ad libitum access to tap water and PK120 laboratory diet (Laboratorkorm Ltd., Moscow, Russia). The animals were daily monitored by a trained veterinary expert. A carbon dioxide chamber was used to sacrifice the animals. Clinical blood tests, undertaken at the time of sacrifice (please, see below), demonstrated the absence of acute toxicity (slight leucopenia and anemia were observed in both cisplatin groups compared to intact control—Appendix A, Table A1).

### 4.2. Study Design

The study was conducted in 24 rats. The animals were randomly assigned for chemoperfusion (HIPEC, 12 animals), intravenous (i.v.) administration (9 animals), and intact control group (3 animals). The animals were divided into the following subgroups:

Three animals from HIPEC groups were used to study the initial phase of platinum pharmacokinetics (platinum accumulation in the bloodstream during and immediately after the perfusion). Sample collection was undertaken at the following time points: −45 (before the perfusion), −30, −15, 0 (end of perfusion), 15, 30, and 60 min after the end of the perfusion.

Three animals for HIPEC and three animals for i.v. administration were used to study the middle section of the pharmacokinetic curve. The sampling was undertaken at 0, 120, 240, 480 min, and 24 h after HIPEC and 15, 30, 60, 120, 240, 480 min, and 24 h after i.v. injection of cisplatin. The animals were sacrificed after 24 h to assess platinum accumulation in the organs.

Three animals per each administration method were used to evaluate the prolonged platinum retention in the bloodstream. The sampling was undertaken before the administration of cisplatin, 15 min after the perfusion, or i.v. administration, and after 1, 2, and 5 days after the administrations. After 5 days, the animals were sacrificed; the blood was collected for hematological tests; ovaries, kidneys, and liver were excised.

Three intact rats (intact control—I.C.) were used as a reference for histological, immunohistochemical, and hematological studies. Blood, kidneys, liver, and ovaries were collected from these animals.

Six animals (3 for HIPEC and 3 for i.v. administration) were euthanized 15 min after the end of HIPEC or i.v. administration of cisplatin. The blood was collected at the time of sacrifice to be used for the quantification of ‘free’ (ultrafiltrable) platinum.

### 4.3. Cisplatin Administration

#### 4.3.1. Chemoperfusion

The perfusion (HIPEC) protocol has been described elsewhere [23]. Briefly, the animals were under general anesthesia; inflow and outflow catheters were inserted into the right upper abdomen and the left lower abdomen, correspondingly. The catheters were attached to the closed perfusion circuit containing ca. 100 mL of saline with cisplatin (Cisplatin-LANS^®^ for infusions 0.5 mg/mL, lot No. 90519, best before May 2022, Veropharm Ltd., Moscow, Russia) in the dose of 20 mg per kg b.w. The temperature of the perfusion solution throughout the whole procedure was maintained at 39.5–40.5 °C.

#### 4.3.2. Intravenous Injection

Cisplatin was administered i.v. into the caudal vein in the dose of 4 mg per kg b.w.

### 4.4. Samples and Sample Handling

#### 4.4.1. Blood Sampling

For platinum quantification, blood was collected in vivo from the caudal vein into the MiniCollect^®^ TUBE 0.25/0.5 mL K_3_EDTA tubes (Greiner Bio-One, Kremsmünster, Austria). Higher volumes of blood for ultrafiltrable platinum assay and hematology were collected during the euthanasia via cardiac puncture into the BD Vacutainer^®^ 4 mL K_3_EDTA tubes (Becton Dickinson, Vianen, The Netherlands). The samples were frozen at −40 °C before further use.

#### 4.4.2. Blood Ultrafiltrate for ‘Free’ Platinum Assay

Whole blood was centrifuged at 2000× *g* for 10 min to collect blood plasma. The obtained plasma was applied to 10 kDa cutoff membranes filters (Amicon^®^ Ultra-4 Centrifugal Filter Units, purchased from Sigma-Aldrich, Merck AG, Darmstadt, Germany) and centrifuged for 10 min at 4000× *g*.

#### 4.4.3. Platinum-Containing Perfusate

The perfusate was collected immediately after the HIPEC sessions. The samples were stored in 1.5 mL Safe-Lock Tubes (Eppendorf, Hamburg, Germany) and frozen at −40 °C before further analysis.

#### 4.4.4. Organs for Platinum Quantification

The following organs were collected for platinum quantification at the time of sacrifice: liver, kidney, and ovary. Half of the liver, left kidney, and ovaries were packed in tight sterile polypropylene bags and frozen at −40 °C before further use.

#### 4.4.5. Samples for Pathological Examination

The rest of the liver and right kidney were fixed in 10% buffered formalin for 48 h. Pieces of kidney and liver were subjected to routine histological processing and mounted in paraffin blocks [46]. A total of 3–5 µm microtome slices were prepared for H&E or immunohistochemical staining.

### 4.5. Quantification of Platinum

#### 4.5.1. Total Platinum Quantification in Blood and Tissue

For platinum quantification, inductively coupled plasma mass spectrometry (ICP-MS) was used as described previously [47,48]. In brief, a microwave system UltraClave™ (Milestone, Sorisole, Italy) was used for sample digestion. Suprapur^®^ nitric acid (65%, Merck, Darmstadt, Germany) and Milli-Q^®^ water (resistance 18.2 MΩ cm, obtained by Milli-Q^®^ Advantage A10, Millipore, Molsheim, France) were used for sample digestion and calibration solution preparation. Samples were thawed at +4 °C and digested with 3 mL 65% nitric acid and 1 mL Milli-Q^®^ water for blood (0.5 mL) and tissue (*ca*. 100 mg), respectively. Microwave treatment was performed for 30 min (maximum autoclave temperature 185 °C, power 1000 W). The resulting solutions were diluted to the final volume of 25 mL in PFA volumetric flasks. At this stage, the internal standard (yttrium) was added to the final concentration of 1 μg/L. An Analytik Jena PlasmaQuant MS Elite (Analytik Jena, Jena, Germany) mass spectrometer with a quartz concentric Micromist nebulizer, +3 °C Peltier-cooled glass spray chamber, and standard nickel cones was used. The following instrument parameters were used: collision mode, helium flow 120 mL/min; plasma parameters: plasma gas flow 9 L/min, auxiliary gas flow 1.35 L/min, nebulizer gas flow 1.08 L/min (daily optimized), plasma RF power 1360 W. The result was calculated as a mean of 9 replicate measurements of the corresponding mass to charge ratio (195 for Pt and 89 for Y, internal standard) unless relative standard deviation (RSD) exceeded 5% [47]. Internal calibration was plotted in the range 1.0–10.0 µg/L. The calibration was established directly before analyzing the samples and its stability was controlled after every 10 samples. The procedure was previously validated using the spiking method in the range 4.1 µg/L (limit of quantification) to 200 µg/L [47]. Additionally, quality controls samples with known concentration of platinum were randomly distributed between the samples under study and analyzed blindly to ensure data quality.

#### 4.5.2. Ultrafiltrable Platinum Quantification

The ultrafiltration system was tested for platinum retention using pure cisplatin solutions with the concentrations of 40 and 7.5 mg/L. The solutions were treated analogously to the plasma samples (Section 4.4.2) using 10 kDa filters (Amicon^®^ Ultra-4). The recovery of over 95% of platinum was observed (Table 3), which is in line with the manufacturer’s information. The lower limit of 96.46% was used for recovery calculations in ultrafiltration studies.

### 4.6. Histology and Immunohistochemistry

#### 4.6.1. Pathological Examination

H&E stained kidney sections were evaluated semi-quantitatively for tubular injury severity by a trained morphologist in a blinded manner. The sections were scaled for tubular necrosis area as following: 0—no pathological changes, 1—<10%, 2—10–20%, 3—20–30%, 4—more than 30% of necrotic area.

#### 4.6.2. Immunohistochemical Anti-H2AX Staining

The slides were deparaffinized. After that, heat-induced antigen retrieval and endogenous peroxidase blocking were consecutively performed. The samples were incubated with primary rabbit (anti-gammaH2AX(p139), ab81299, purchased from Abcam, Cambridge, MA, USA) and secondary anti-rabbit antibodies (ab214882, Abcam) for 1 h at room temperature. The incubation was followed with triple tris-saline buffer (pH 7.6) washes. Diaminobenzidine (DAB) was used as a chromogen. The images were acquired using a Nikon Eclipse Ni-U microscope equipped with a Nikon Digital Sight DS-Fi2 camera. The analysis of specific staining was performed using ImageJ software (NIH, Bethesda, MD, USA).

To assess DNA damage, the nuclei were divided in 5 categories: 0—no visible staining, 1—one or several small foci, 2—more than 3 foci or weak staining of most chromatin, 3—moderate staining of most or total chromatin, 4—intense staining. A trained pathologist assessed the slides in a blinded manner. DNA damage index was calculated for each photomicrograph as follows: $DNADI,\;\%=\left(0n_{1}+1n_{2}+2n_{3}+3n_{4}+4n_{5}\right)/N$, where $n_{0}-n_{4}$ is the number of nuclei of each type, *N* is the sum of all scored nuclei. The damage in study groups was compared to the baseline level in I.C.; the baseline DNA damage is constantly present in the metabolically active hepatocytes and other cells mainly due to the formation of reactive oxygen and nitrogen species [49].

### 4.7. Statistics

Mann–Whitney U rank test was used to compare the values. DNA damage index data were analyzed using a non-parametrical Kruskal–Wallis H rank test. The statistical level of *p* < 0.05 was considered as significant. Prism 6 (GraphPad Software, San Diego, CA, USA) was used for calculations. AUC was evaluated by a model-independent approach using trapezoid rule.

## Figures and Tables

**Table 1:** Retention of cisplatin-derived Pt in rat body after HIPEC. Calculated as average for 12 rats (*n* = 12).

	Body Weight, g	Volume of Perfusate, mL	Initial Pt in Perfusate, mg/L	Final Pt in Perfusate, mg/L	Retained Pt in Rats, mg/kg
Average	250.00	93.67	34.75	21.28	5.01
SD	17.00	7.78	2.16	1.23	0.44

**Table 2:** The concentrations of “free” (10 kDa ultrafiltrable) platinum after HIPEC and i.v. injections of cisplatin in rats 15 min after the injection. Calculated as average for three rats (*n* = 3).

Injection Route/Dose	HIPEC, Cisplatin 20 mg/kg b.w.	i.v., Cisplatin 4 mg/kg b.w.
Blood Pt conc., mg/L ± SEM	2666 ± 266	1051 ± 80
Plasma Pt conc., mg/L ± SEM	2653 ± 277	972.9 ± 45.9
Filtrate Pt conc., mg/L ± SEM	1310 ± 117	682.1 ± 21.3
Permeability, % ± SEM	53.08 ± 0.92	73.76 ± 1.32 *
Binding, % ± SEM	46.92 ± 0.92	26.24 ± 1.32 *

*—*p* < 0.001 (Mann–Whitney U rank test).

**Table 3:** Ultrafiltration system recovery (*n* = 3).

Model Solution Used	Cisplatin, 40 mg/L	Cisplatin, 7.5 mg/L
Initial solution Pt conc., mg/L ± SEM	25.49 ± 0.33	4.89 ± 0.07
Filtrate Pt conc., mg/L ± SEM	24.90 ± 0.09	4.70 ± 0.05
Recovery, % ± SEM	97.70 ± 0.92	96.46 ± 0.29

## Appendix A

**Table A1:** Hematological parameters in blood of animals at 5th day of experiment. Calculated as average for 3 rats (*n* = 3) ± SEM.

Parameter	WBC 10^9^/L	Lymph 10^9^/L	Mon 10^9^/L	Gran 10^9^/L	RBC 10^12^/L	HGB g/L	PLT 10^9^/L
I.C.	16.8 ± 0.7	12.6 ± 1.1	0.50 ± 0.06	3.70 ± 0.78	9.36 ± 0.05	157.3 ± 4.3	905.3 ± 324.1
HIPEC	12.2 ± 2.0	8.3 ± 1.0 *	0.37 ± 0.09	3.57 ± 0.92	7.44 ± 0.16 *	129.7 ± 3.9	591.7 ± 257.3
I.V.	11.5 ± 1.5	8.2 ± 1.1 *	0.27 ± 0.03*	3.00 ± 0.38	7.07 ± 0.34 *	124.7 ± 10.8	524.7 ± 300.2

*—*p* < 0.05 (Student *t*-test). HIPEC—hyperthermic intraperitoneal chemoperfusion of cisplatin (20 mg/kg b.w.); I.C.—intact control; I.V.—intravenous administration of cisplatin (4 mg/kg b.w.); WBC—white blood cell count; Lymph—lymphocyte count; Mon—monocyte count; Gran—granulocyte count; RBC—red blood cell count; HGB—hemoglobin concentration; PLT—platelet count.
